# From Gut to Globe: Endogenous Endophthalmitis as a Sentinel Sign of Polymicrobial Intra‐Abdominal Infection

**DOI:** 10.1002/ccr3.71946

**Published:** 2026-01-29

**Authors:** Dhruv Sethi, Bilal Khan, Bushra Rahman, Arslon Humayun, Osama Ahmed, David Goldman, Mohsin H. Ali

**Affiliations:** ^1^ Retina Group of Washington Fairfax Virginia USA; ^2^ School of Medicine Georgetown University Washington DC USA; ^3^ Henry Ford Health System Detroit Michigan USA

**Keywords:** endogenous endophthalmitis, hepatic abscess, *Klebsiella pneumoniae*, polymicrobial bacteremia, vision loss

## Abstract

A 42‐year‐old male presented with 1 week of left eye redness, pain, and severe vision loss. Examination revealed anterior chamber fibrin, vitritis, and vitreous opacities. B‐scan ultrasonography showed mobile hyperechoic vitreous material with an attached retina. Given his recurring fevers, a vitreous tap and injection of intravitreal antibiotics was performed, followed by systemic infectious workup. Blood cultures revealed polymicrobial bacteremia with *Klebsiella pneumoniae* and Salmonella serogroup B, secondary to colonic and hepatic abscesses confirmed by CT. Despite treatment, the eye progressed to phthisis bulbi. This case underscores the importance of ocular symptoms in identifying serious systemic infections.

## Key Clinical Message

Endogenous endophthalmitis may be the first and only sign of a life‐threatening systemic infection, even in immunocompetent individuals. This case highlights the importance of prompt ophthalmic evaluation and systemic workup, particularly when vision‐threatening inflammation is present in the absence of overt systemic illness.

## 1 Introduction

Endogenous endophthalmitis is a rare but potentially devastating intraocular infection caused by hematogenous spread from a distant systemic source, accounting for 5%–15% of endophthalmitis cases [1]. It typically arises in the setting of systemic infections such as endocarditis, urinary tract infections, or hepatic abscesses, and is most often caused by gram‐positive cocci, gram‐negative bacilli, or fungi.

Culture‐positive endophthalmitis studies consistently show gram‐positive organisms as the most common isolates overall, with gram‐negative pathogens representing an important subset associated with severe or fulminant presentations [2]. In Western countries, *Klebsiella pneumoniae* has emerged as a notable cause, while Salmonella is exceedingly rare, especially in immunocompetent adults. Endogenous endophthalmitis cases portend significantly poor visual outcomes, including an associated mortality rate of 5% due to extraocular foci of infections [3].

We describe a previously healthy man who developed endogenous endophthalmitis from polymicrobial bacteremia with *Klebsiella pneumoniae* and Salmonella serogroup B. His presentation highlights the need to consider systemic infection in cases of intraocular inflammation, even in patients without significant immunosuppression or overt systemic signs.

## 2 Case History/Examination

A 42‐year‐old male presented to the retina clinic with 1 week of left eye redness, mild pain, and decreased vision. His history included remote abdominal trauma following an assault, requiring exploratory laparotomy and splenectomy, placing him at increased lifelong risk for severe infections with encapsulated organisms. He reported subjective fevers and chills over the prior 3 weeks. COVID‐19 testing ordered by his primary care physician was negative.

**FIGURE 1:**
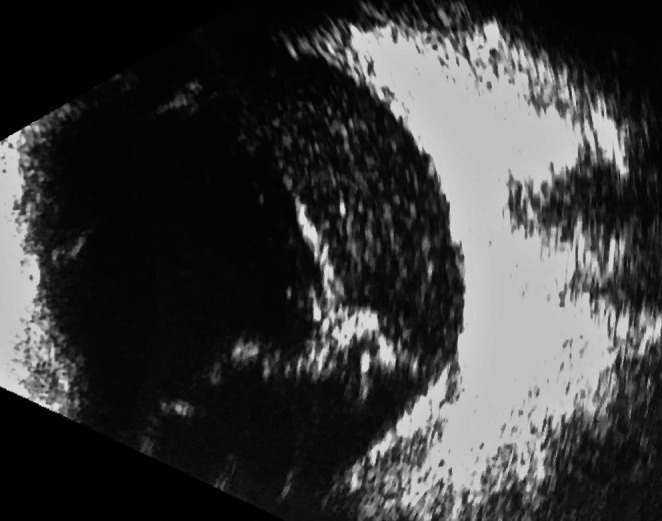
B‐scan ultrasonography of the left eye. B‐scan ultrasonography revealed mobile hyperechoic vitreous opacities and membrane formation.

On exam, visual acuity was 20/20 OD and light perception OS, with a left relative afferent pupillary defect. Intraocular pressures and motility were normal bilaterally. The right eye was unremarkable. The left eye showed 3+ conjunctival injection, 4+ fibrin with hemorrhage in the anterior chamber, dilated iris vessels, and 3+ nuclear sclerosis. Fundus view OS was obscured by 4+ vitritis and dense vitreous opacities. B‐scan ultrasonography revealed dense, mobile hyperechoic vitreous material, mild choroidal thickening, membrane formation, and an attached retina (Figure 1). The rest of the ophthalmic exam was unremarkable.

## 3 Differential Diagnosis, Investigations, and Treatment

With an exam diagnostic of endophthalmitis, and no exogenous source that could be readily attributed, a workup for endogenous sources was arranged. At this time, a vitreous tap was performed and sent for bacterial and fungal cultures. Intravitreal injections of vancomycin 1 mg/0.1 mL and ceftazidime 2.25 mg/0.1 mL were administered (Figure 1).

**FIGURE 2:**
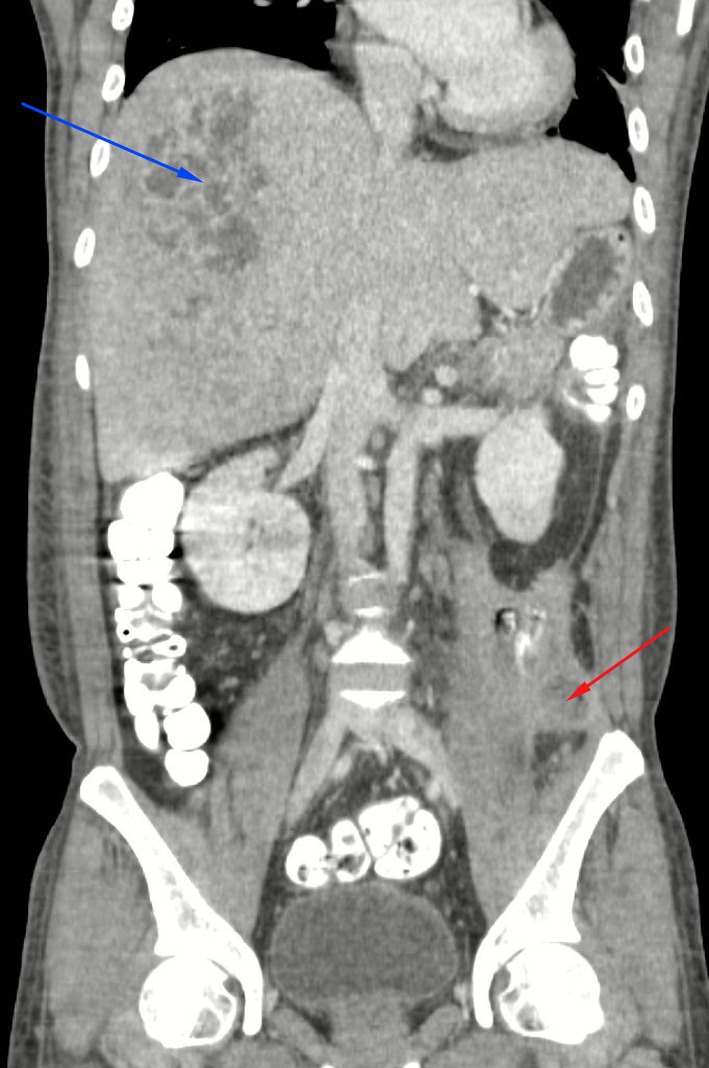
Computed tomography with oral and intravenous contrast of the abdomen and pelvis (coronal scan). Hypodense heterogeneous lesions corresponding to a large hepatic abscess (blue arrow) and pericolonic abscess (red arrow) were detected and subsequently treated with percutaneous drainage.

Blood cultures grew *Klebsiella pneumoniae* and Salmonella serogroup B, while echocardiogram was unremarkable. CT abdomen and pelvis revealed colonic perforation with a pericolonic abscess and a large hepatic abscess (Figure 2), both of which were drained percutaneously. The patient was admitted and underwent systemic inpatient treatment for sepsis including intravenous antibiotics. During follow up, his ophthalmic exam worsened with progression to pre‐phthisis and no light perception (NLP). At this time, given the vision loss and concurrent management of systemic comorbidities, goals of treatment evolved to comfort care with no further injections or surgical treatment (Figure 2).

## 4 Conclusion and Results

This patient's anterior chamber inflammation and vitritis, along with systemic symptoms, had raised early suspicion for endogenous endophthalmitis. In response, intravitreal vancomycin and ceftazidime were administered. While vitreous cultures were negative for bacterial and fungal growth, systemic workup revealed Klebsiella and Salmonella bacteremia with underlying colonic perforation, pericolonic abscess, and hepatic abscess. The left eye progressed to phthisis despite therapy, likely reflecting the fulminant nature of the polymicrobial infection in the setting of a delayed presentation before initiating management.

## 5 Discussion

Endogenous endophthalmitis overall is uncommon, comprising less than 2%–8% of endophthalmitis cases, and often carries a poor prognosis [4]. Common sources of infection include soft tissue infections, endocarditis, meningitis, urinary tract infections, indwelling catheters, and IV drug use [1]. In East Asia, Klebsiella‐associated liver abscesses account for the majority of cases, including a report by Li et al. [5] describing a case of Klebsiella liver abscess complicated by meningitis and endogenous endophthalmitis. However, our patient's case was polymicrobial, stemming from gastrointestinal perforation secondary to an unusual combination of Salmonella and Klebsiella coinfection.

Polymicrobial endogenous endophthalmitis is associated with extremely poor visual prognosis. Eyes presenting with light perception vision and dense fibrin often fail to respond to medical or surgical intervention [6]. Likely, Salmonella gastroenteritis led to pericolonic abscess and colonic perforation, allowing polymicrobial bacteremia and subsequent Klebsiella co‐infection with hepatic abscess and ocular seeding (Figure 2). Systemic symptoms were mild despite the severity of infection, and without prominent ocular signs, the diagnosis may have been delayed.

Salmonella‐associated endophthalmitis is rarely documented but can occur regardless of immune status [4, 7]. Although otherwise immunocompetent, our patient's asplenic status likely increased his susceptibility to invasive infection, as the spleen plays a critical role in clearing encapsulated bacteria such as *Klebsiella pneumoniae* and Salmonella species. Polymicrobial endophthalmitis is also rare; in a report by Jindal et al. of 1107 cases, only 3.88% were polymicrobial, and only three cases were endogenous in etiology [8]. Other reports describe Salmonella‐related endophthalmitis following gastrointestinal illness, reinforcing that even subtle systemic symptoms may precede vision‐threatening disease [9]. Klebsiella's role in endogenous endophthalmitis also appears to have an increasing global relevance [5, 10]. With regard to *Klebsiella pneumoniae* pyogenic liver abscesses alone, a 2020 systematic review and meta‐analysis of 11,889 patients across fifteen retrospective studies found a 4.5% pooled incidence of endogenous endophthalmitis [11].

In this patient, polymicrobial bacteremia from abdominal abscesses led to vision loss. Despite treatment with intravitreal and intravenous antibiotics with percutaneous drainage, vision unfortunately deteriorated to no light perception, and the eye developed phthisis bulbi.

This case illustrates that endogenous endophthalmitis may be an initial clue to life‐threatening systemic infection. Clinicians should consider systemic workup in patients with severe intraocular inflammation, even without overt systemic signs. Early recognition of endogenous endophthalmitis is essential to preserve vision and life.

## Author Contributions

**Dhruv Sethi:** investigation, writing – original draft, writing – review and editing. **Bilal Khan:** investigation, writing – original draft, writing – review and editing. **Bushra Rahman:** investigation, writing – review and editing. **Arslon Humayun:** investigation, writing – review and editing. **Osama Ahmed:** writing – review and editing. **David Goldman:** writing – review and editing. **Mohsin H. Ali:** conceptualization, investigation, methodology, supervision, writing – original draft, writing – review and editing.

## Funding

The authors have nothing to report.

## Ethics Statement

The authors have nothing to report.

## Consent

Written informed consent and permission for publication was obtained.

## Conflicts of Interest

The authors declare no conflicts of interest.

## Data Availability

The data that support the findings of this study are available on request from the corresponding author. The data are not publicly available due to privacy or ethical restrictions.
